# Supplementary material for: MetaRibo-Seq measures translation in microbiomes
Source: Nat Commun. 2020 Jun 29;11:3268. doi: 10.1038/s41467-020-17081-z (PMC7324362; doi:10.1038/s41467-020-17081-z)
Supplement: Supplementary file 10 — Supplementary Data 7 [file 41467_2020_17081_MOESM10_ESM.zip › File2/Confidence_High_Taxonomy/276980_out.krona.html]

Javascript must be enabled to view this page.

members
magnitude
magnitudeUnassigned
count
unassigned
taxon
rank

276980\_out

5

2759

SRS013687\_contig\_number\_8723
1
superkingdom
5

4751
4
kingdom

451864
4
subkingdom

4890
phylum
4

subphylum
4
147538

147550
class
4

4
subclass
222543

5125
4
order

110618
4
family

5506
genus
4

species

SRS023914\_contig\_number\_contig-100\_431.160777SRS051031\_contig\_number\_contig-100\_347.199303SRS076929\_contig\_number\_contig-100\_236.152239SRS1041145\_contig\_number\_37775
4
1803897
